# Local Inflammatory and Systemic Antibody Responses Initiated by a First Intradermal Administration of Autogenous *Salmonella*-Killed Vaccines and Their Components in Pullets

**DOI:** 10.3390/vaccines12101159

**Published:** 2024-10-11

**Authors:** Jossie M. Santamaria, Chrysta N. Beck, Gisela F. Erf

**Affiliations:** Department of Poultry Science, Center of Excellence for Poultry Science, University of Arkansas System Division of Agriculture, Fayetteville, AR 72701, USA; cnbeck@uark.edu

**Keywords:** local cellular immune response, antibody response, *Salmonella* vaccines, lipopolysaccharide, emulsion, pullets

## Abstract

Vaccination strategies are used to manage *Salmonella* in chickens. *Salmonella*-killed vaccines are considered safer since they are inactivated. However, little is known regarding the cellular immune activities at the site of vaccine administration of *Salmonella*-killed vaccines. The growing feather (GF) cutaneous test has been shown to be an effective bioassay to monitor local tissue/cellular responses. We assessed local and systemic antibody responses initiated by intradermal injection of *Salmonella*-killed vaccines into GF-pulps of 14–15-week-old pullets. Treatments consisted of two autogenous *Salmonella*-killed vaccines (SV1 and SV2), *S.* Enteritidis (SE) lipopolysaccharide (SE-LPS), and the water-oil-water (WOW) emulsion vehicle. GF-pulps were collected before (0 h) and at 6, 24, 48, and 72 h post-GF-pulp injection for leukocyte population analysis, while heparinized blood samples were collected before (0 d) and at 3, 5, 7, 10, 14, 21, and 28 d after GF-pulp injections to assess plasma levels (a.u.) of SE-specific IgM, avian IgY (IgG), and IgA antibodies using an ELISA. Injection of GF-pulps with SV1, SV2, or SE-LPS, all in a WOW vehicle, initiated inflammatory responses characterized by the recruitment of heterophils, monocytes/macrophages, and a few lymphocytes. The WOW vehicle emulsion alone recruited more lymphocytes than vaccines or SE-LPS. The SV1 and SV2 vaccines stimulated *Salmonella*-specific IgM and IgA early, while IgG levels were greatly elevated later during the primary response. Overall, SV1 and SV2 stimulated a heterophil and macrophage-dominated local inflammatory- and SE-specific humoral response with an isotype switch from IgM to IgG, characteristic of a T-dependent primary antibody response. This study provides comprehensive information on innate and adaptive immune responses to autogenous *Salmonella*-killed vaccines and their components that will find application in the management of *Salmonella* in poultry.

## 1. Introduction

*Salmonella* Enteritidis (SE) is one of the most prevalent serovars involved in the contamination of chicken products that cause human infection via the consumption of inadequately cooked chicken meat and eggs [1,2,3,4]. Because adult chickens with non-typhoidal *Salmonella* infection do not exhibit signs of illness, they can remain carriers, releasing the bacteria into the environment through their feces [3]. The poultry industry aims to decrease *Salmonella* colonization and fecal shedding to reduce vertical transmission and environmental contamination. Various approaches have been developed to manage *Salmonella* infection in poultry flocks to safeguard people from *Salmonella* food poisoning [3,5,6,7,8]. Among various preventative measures, vaccination against *Salmonella* is one of the most promising approaches to manage *Salmonella* contamination of food products [7,9,10]. *Salmonella* colonization in chickens is controlled primarily using three different vaccine types: live attenuated, subunit, and inactivated (killed) vaccines [11]. Combinations of live-plus-inactivated vaccination regimens are becoming more popular due to a need to extend the withdrawal period between live vaccine administration and the pullet’s point of lay [9,12]. Additionally, there remains a significant concern with live attenuated vaccines due to their potential return to bacterial virulence [8,10,13]. Inactivated bacterial vaccines (bacterins) cannot multiply and, therefore, cannot return to virulence, making them a safer option. Among bacterins, autogenous *Salmonella* inactivated vaccines are commonly used to target a novel variant of the pathogen effectively; they are customized by isolating and inactivating flock-specific, problematic *Salmonella* serotypes and can be produced in a short time in response to an increase in pathogen prevalence or outbreaks [14,15]. In broiler breeder and layer pullets, different health issues have been associated with the administration of *Salmonella* bacterin vaccines, such as severe local inflammatory reactions at the injection site and vaccine-associated hemorrhagic hepatitis that could lead to mortality ranging between 0.2% and 5% [16,17]. Most commercial autogenous *Salmonella* bacterin vaccines contain oil-based adjuvants, and their administration has been linked to the formation of oily cysts, granulomas, and sometimes tissue necrosis [16,18]. There is little information regarding the cellular/tissue immune responses at the site of administration and the associated humoral responses to autogenous *Salmonella* bacterin vaccines.

The growing feather (GF-) pulp cutaneous test system is a novel, minimally invasive, in vivo method to assess and monitor local cellular/tissue responses at the site of intradermal (i.d.) GF-pulp injection of test materials in chickens [19,20]. Briefly, simultaneous i.d. pulp injection of test material into multiple GFs of an individual and periodic collection of GFs post-injection for ex vivo laboratory analyses have been a successful biopsy-like approach to gain insights into immune responses to the test material in a complex tissue. This approach has been successfully used to demonstrate the time-course as well as quantitative and qualitative aspects of in vivo cellular/tissue responses at the site of injection to microbes and microbial components [20,21]. Due to the minimally invasive nature of the GF test system, systemic responses to the i.d. pulp injections may be monitored in the same individual by concurrent sampling of peripheral blood. This dual-window approach offers a valuable tool for monitoring and assessing local cellular and systemic humoral immune responses to vaccine treatments.

The objective of this study was to examine the tissue/cellular immune activities at the site of i.d. vaccine administration (GF-pulp) and the resulting systemic (blood) antibody responses to a first immunization with *Salmonella* bacterin vaccines and components in 14–15-week-old Light brown Leghorn (LBL) pullets. Specifically, treatments consisted of two autogenous *Salmonella* bacterin vaccines (SV1 or SV2), pure *S*. Enteriditis LPS, and the water-oil-water (WOW) emulsion vaccine vehicle. We monitored and assessed the local leukocyte infiltration profiles (monocytes/macrophages, heterophils, T cells, and B cells) in GF-pulps over a 72 h time period, as well as the SE-specific antibody response profiles, including IgM, avian IgY (IgG), and IgA in the peripheral blood over 4 weeks post-GF-pulp injection of the four test materials.

## 2. Materials and Methods

### 2.1. Experimental Animals

The University of Arkansas System Division of Agriculture Animal Care and Use Committee (UADA-IACUC) authorized all protocols and procedures that involved animals in this experiment (UADA-IACUC approval number 21035). Three replicate Trials were conducted involving 14–15-week-old Light-brown Leghorn (LBL) pullets from the LBL breeder population maintained by Dr. Erf’s research group at the UADA Poultry Research Farm in Fayetteville, AR. Trial 1 consisted of 14 pullets, while Trials 2 and 3 had 15 pullets each. Chicks were hatched at the UADA Poultry Research Farm’s hatchery and tagged with identification numbers. For Trial 3, chicks were also vaccinated subcutaneously with live herpesvirus of turkey (HVT) Marek’s disease vaccine (MD Vac, Zoetis, Parsippany, NJ, USA) at hatch. For Trials 1 and 2, straight run chicks were placed on wood shaving litter in floor pens at the UADA Poultry Health Laboratory (PHL), Fayetteville, AR, whereas for Trial 3, chicks were reared in floor pens on wood shaving litter at the UADA Poultry Research Farm (Farm). Chicks for Trials 2 and 3 were from the same hatch. For all Trials, standard rearing temperature, diet, and lighting conditions were followed. Feed and water were available ad libitum, and the well-being of chickens was checked daily [20,21]. For all Trials, females were separated from males and placed into separate pens (6 ft by 3.5 ft, 8 females per pen) at 8 weeks of age.

### 2.2. Test Materials

Two different autogenous, formalin-killed *Salmonella* bacterin vaccines (SV1 and SV2) and a water-oil-water (WOW) emulsion (vehicle) were obtained from Elanco Animal Health (Greenfield, IN, USA). Both SV1 and SV2 autogenous bacterin vaccines contained 4 serovars, with *S*. Enteritidis being the dominant serovar in both vaccines. Although both SV1 and SV2 were prepared at a concentration of 10^8^ bacterial cells/mL, the endotoxin content was higher in SV1 than in SV2 (180,000 versus 68,000 EU/mL, respectively). *S*. Enteritidis LPS (SE-LPS; Sigma, Saint Louis, MO, USA, L6011) was prepared in the WOW vehicle at 180,000 EU/mL by Elanco Animal Health. In addition to stock materials, doubling dilutions of SV1 and SV2 (1 in 2 and 1 in 4) and SE-LPS (1 in 5 and 1 in 10) were aseptically prepared in a biosafety cabinet (Thermo Scientific 1300 Series A2, Waltham, MA, USA) by mixing the stock with appropriate volumes of the vehicle. To ensure homogenous mixing, the stock material and the WOW emulsion vehicle were placed in a 10 mL syringe, and the materials were pushed back and forth 20 times between two 10 mL syringes connected by a syringe coupler adaptor (Dispense All Syringe Adaptors, C-U Innovations, LLC, Chicago, IL, USA). The undiluted and diluted materials were designated as high (H), medium (M), and low (L) concentrations of test materials (e.g., SV1-H, SV1-M, and SV1-L for the undiluted, the 1 in 2, and 1 in 4 dilutions of SV1, respectively).

### 2.3. Experimental Trials

#### 2.3.1. Trial 1

Trial 1 was initially designed the same as Trials 2 and 3, described below. However, the approach needed to be simplified due to COVID-19 laboratory access restrictions. Hence, for Trial 1, the pulps of 14 18-day regenerating GF per pullet were i.d. injected with either SV1-H, SV2-H, or SE-LPS-H (10 µL per GF; 4 pullets per treatment). Additionally, two pullets from each treatment group had 4 GF injected with vehicle (V). One GF was collected from each chicken before (0 h) and at 6, 24, 48, and 72 h post-GF-pulp injection. Due to restricted laboratory access, same-day preparation of pulp cell suspensions and cell population analyses of the collected GFs could not be conducted on these samples. Blood was collected from the wing vein using heparinized 3 mL syringes with 25 × 1-inch needles (Becton Dickinson, Franklin Lakes, NJ, USA) before (0 day) and at 3, 5, 7, 10, 14, 21, and 28 d post-i.d. GF-pulp injection of the test materials. Before returning the bird to its pen, the bleeding was fully stopped. The use of the left- and right-wing veins was alternated between sampling times. Plasma was separated from whole blood, frozen, and stored at −80 °C until the determination of relative circulating levels of SE-specific IgM, IgG, and IgA using an ELISA.

#### 2.3.2. Trials 2 and 3

For Trials 2 and 3, 14- to 15-week-old pullets were randomly assigned to SV1, SV2, SE-LPS, and V treatments; 4 birds each for SV1, SV2, and SE-LPS treatments, and 3 birds for V control treatment. For SV1, SV2, and SE-LPS treatments, 12 GF on the left breast tract were injected with the high dose, and on the right breast tract, 6 GF were injected with the medium dose and 6 GF with the low dose. For the control group, 12 GF on the left and right breast tracts were injected with V. All injections consisted of 10 µL of test materials per GF.

GFs were collected from the experimental animals before (0 h) and 6, 24, 48, and 72 h following GF-pulp injection of the test materials. At each time point, one of each GF injected with an H, M, or L dose of the test material was collected from a pullet, placed in separate tubes with ice-cold PBS, and kept on ice until the processing of pulp cell suspensions. Heparinized blood (1.5 mL) was collected before (0 d) and at 3, 5, 7, 10, 14, 21, and 28 d post-pulp injection as described for Trial 1. Plasma was separated from whole blood, frozen, and stored at −80 °C until the determination of circulating levels of SE-specific IgM, IgG, and IgA using an ELISA.

### 2.4. Preparation and Immunofluorescent Staining of Pulp Cell Suspensions and Cell Population Analysis by Flow Cytometry

Pulp cell suspensions of the sampled GF from Trials 2 and 3 were prepared and immunofluorescently stained as described previously [21]. Briefly, the entire pulp of a GF was extracted from the sheath and placed in 1 mL of a 0.1% collagenase (type IV, Life Technologies, Carlsbad, CA, USA) and 0.1% dispase II (Boehringer Mannheim, Mannheim, Germany) PBS solution, and incubated at 40 °C for 10 min. Following incubation, the pulps were gently pushed through a 60 μm nylon mesh while ice-cold PBS was added. The pulp cell suspensions were washed twice in PBS at 250× *g* for 8 min at 4 °C, and the pellet was resuspended in PBS+ (PBS, 1% bovine serum albumin, and 0.1% sodium azide). Using fluorescently labeled mouse monoclonal antibodies (mAb) specific for chicken leukocyte markers (Southern Biotech, Birmingham, AL, USA), leukocytes in the pulp cell suspensions were identified by two-color direct immunofluorescently staining as described previously [21,22]. Specifically, cell suspensions were dual labeled with either a mixture of mAb specific for the chicken pan-leukocyte marker CD45 conjugated to spectral red fluorochrome (CD45-SPRD) together with a phycoerythrin (PE)-conjugated mAb specific for the chicken monocyte/macrophage marker KUL01 (KUL01-PE) or a mixture of a mAb specific for the chicken B cell marker Bu-1 conjugated to PE (Bu-1-PE) and mAb specific to the chicken pan-T cell marker CD3 (CD3-SPRD). All mouse mAbs utilized had the IgG1 isotype. PE- and SPRD-conjugated IgG1 isotype controls (Southern Biotech) were used to test for non-specific binding of leukocyte-specific mAbs, and single-stained populations were used to set compensation. After a 30 min incubation, the plates were centrifuged twice at 250× *g* for 4 min at 4 °C, and the supernatant fluid was discarded. Cell pellets were then resuspended in 100 μL of PBS and fixed by adding another 100 µL of a 1% paraformaldehyde solution. Plates were stored at 4 °C for 3–5 days until cell population analysis using fluorescence-based flow cytometry. Prior to flow cytometric cell population analysis, plates were washed at 250× *g* for 4 min at 4 °C, and the supernatant fluid was discarded. Cells were resuspended in 200 µL of PBS, and the samples were subjected to two-color cell population analysis using a BD FACSAria Fusion (Becton Dickinson Immunocytometry Systems, San Jose, CA, USA) flow cytometer and FlowJo software v10.10 (Flow Jo, LLC, Ashland, OR, USA). The heterophil (avian neutrophil) population was identified based on size (FSC) and granularity (SSC) characteristics of CD45+ leukocytes [23]. All data were expressed as the percentage of stained cells in the total pulp cell suspension (% pulp cells).

### 2.5. Enzyme-Linked Immunosorbent Assay (ELISA) Procedure

Relative levels of circulating SE-specific IgM, IgG, and IgA antibodies were determined using an ELISA, following a previously described procedure with modifications for optimization [24]. Briefly, formalin-killed *S*. Enteritidis (SE) cells were suspended in coating buffer (0.05 M carbonated buffer, pH 7.4) at 1 × 10^7^ CFU/mL, and 100 µL of the SE suspension was added to each well of a 96-well flat-bottomed plate (Thermo Scientific, Waltham, MA, USA, 262146). To allow the cells to adhere, the covered plates were incubated at 37 °C for 2 h (Isotemp incubator, Fisher Scientific, Waltham, MA, USA) followed by overnight incubation at 4 °C. After incubation, the SE-coated wells were washed three times for 2 min each with a washing solution (50 mM Tris, 0.14 M NaCl, 0.05% Tween 20; pH 8.0). Then, the wells were filled with 200 µL of blocking buffer (50 mM Tris, 0.14 M NaCl, 1% BSA, pH 8.0) and incubated for 30 min at RT. After the blocking step, the plates were washed as before. Plates were then designated for an IgM, IgG, or IgA assay. Frozen plasma samples were thawed and diluted in sample diluent buffer [50 mM Tris, 0.14 M NaCl, 1% BSA (bovine serum albumin), 0.05% Tween 20, and pH 8.0] as follows: 1/100 for the IgA assay, and 1/1000 for the IgM and IgG assays. Samples were added to three wells each (100 μL/well) in the IgM, IgG, and IgA assay plates. To create a standard curve, six doubling dilutions of a pool of plasma samples with high SE-specific antibody levels were included in all assays (i.e., plasma pool dilutions were 1/400 to 1/12,800 for IgG and IgM and 1/100 to 1/3200 for IgA assay plates). In addition to the pooled sample, which served as positive controls and the basis for a relative standard curve, other controls added to each plate included a “Blank”, which was set up in triplicate wells, adding the highest concentration of pooled samples for the IgM, IgG, and IgA assays, and a non-specific binding control, adding 100 μL of sample dilution buffer to triplicate wells in place of plasma.

Once all samples and controls were added, the plates were wrapped in aluminum foil and incubated for 2 h at 37 °C. The plates were washed as before, and detection antibodies were added. Detection antibodies, specifically horse-radish-peroxidase (HRP)-conjugated, affinity-purified goat anti-chicken IgG (Ig γ-heavy chain), IgM, and IgA detection antibodies (Bethyl Laboratories, Montgomery, TX, USA), were prepared in sample dilution buffer at 1/20,000 for IgM and IgG, and 1/10,000 for IgA detection. The detection antibodies were added to each well (100 μL/well) in their respective ELISA plates except for the Blank wells, which received unconjugated goat anti-chicken IgM, IgG, and IgA antibodies instead. The plates were then incubated for 1 h at 37 °C and washed (3 × 2 min). A total of 100 µL of TMB substrate solution (3,3′,5,5′-Tetramethylbenzidine one component substrate E102, Bethyl Laboratories) was added to all wells, and plates were incubated at 37 °C for 15 min. The color change reaction was stopped by adding 100 µL of 2M sulfuric acid solution to each well. Color intensity generated by the HRP conversion of the colorogenic substrate was measured (absorbance units; a.u.) at 450 nm in a 96-well spectrophotometer (ELx 800, Biotek, Winooski, VT, USA). The a.u. measurements for the pooled sample dilutions were used to create a standard curve for each plate, and the line equation was used to adjust relative levels (a.u.) of IgM, IgG, and IgA in the plasma for each assay.

### 2.6. Statistical Analyses

All data were analyzed as previously described [21,22] using JMP Pro 16 software (SAS Institute INC., Cary, NC, USA). For Trials 2 and 3, a three-way ANOVA was conducted to determine the main effects of treatment (SV1, SV2, SE-LPS, and V), dose (H, M, and L), and time (0, 6, 24, 48, and 72 h), and their interactions on the levels (% of pulp cells) of various leukocyte populations in GF-pulps. Based on these three-way ANOVAs, there was no main effect of dose and no interactions; hence, for each treatment and time point, the average of the three dose estimates of each pullet was used for further analysis. To assess the main effects of the Trial (i.e., for GF-data Trial 2 and Trial 3), treatment, time, and their interactions on GF-leukocyte profiles, a three-way ANOVA was conducted. In the absence of interactions, data were combined across Trials for a two-way ANOVA to determine the effects of all treatments, time, and their interactions. For plasma antibody response data, a three-way ANOVA examining the effects of trial (Trial 1–3), treatment (SV1, SV2, SE-LPS, and V), and time (0, 3, 5, 7, 10, 14, 21, and 28 d post-i.d. GF-pulp injection) revealed no interactions involving the effects of Trial. Hence, data from Trials 1, 2, and 3 were combined and analyzed using a two-way repeated measure (RM)-ANOVA to determine the effect of treatments, time, and their interactions. Differences between multiple means were tested using the student’s *t*-test as appropriate. In all cases, statistical significance was determined at *p* ≤ 0.05.

## 3. Results

### 3.1. Leukocyte Population Profiles in the Pulp of Growing Feathers before and after Administration of Salmonella Vaccines (SV1 or SV2), SE-LPS, or Vehicle

A three-way ANOVA examining the effects of treatment, time, trial, and their interactions on leukocyte infiltration revealed no interactions involving “Trial” except for T cells. Hence, for all leukocyte populations, except T cells, data were pooled across Trials 2 and 3 and examined using a two-way ANOVA for the effects of treatment, time, and treatment-by-time interactions.

There was a main effect of time (*p* < 0.001) for leukocyte infiltration, where the main effect means ± SEM levels (% pulp cells) increased from 5.4 ± 2.22% at 0 h to maximal levels (38.7 ± 1.63%) at 6 h (*p* < 0.001), then dropped (*p* < 0.001) to 29.6 ± 1.63% at 24 h and continued to decrease (*p* < 0.001) to above pre-injection levels at 48 and 72 h (18.2 and 15.0 ± 1.63%, respectively). There were no differences between treatments and trials for leukocyte infiltration (Table 1).

Chicken leukocytes and macrophages in GF-pulp cell suspensions were identified by direct immunofluorescent staining with mouse-anti-chicken (m-a-c) CD45 or m-a-c KUL01 mouse monoclonal antibodies, respectively, and cell population analysis performed by fluorescence-based flow cytometry. Heterophils were identified based on size (FSC) and granularity (SSC) characteristics of leukocytes (CD45+ cells). Data were expressed as the % of a cell type in the total pulp cell suspension.

Data are the main effect means ± SEM for each time-point (*n* = 14; for each treatment SV1, SV2, and SE-LPS *n* = 40, for V *n* = 30; for each trial and time-point, *n* = 4 birds per treatment (SV1, SV2, and SE-LPS), and *n* = 3 for vehicle control).

Similarly, heterophil main effect mean levels increased from 1.34 ± 1.49% before injection (0 h) to maximal levels (20.1 ± 1.09%) at 6 h (*p* < 0.001) and dropped (*p* < 0.001) to 13.7 and 7.01 ± 1.09% at 24 h and 48 h, respectively. At 72 h, heterophil levels were not different from 48 h but higher (*p* < 0.001) than before injection. Heterophil levels were higher (*p* < 0.013) with SV1, SV2, and SE-LPS treatments (main effect means ± SEM 11.0, 9.77, and 11.4 ± 0.87%, respectively) than with V (5.93 ± 0.87%), and not different from each other. Furthermore, independent of treatment and time, heterophil levels differed between trials (*p* = 0.022), with overall higher levels (10.7 vs. 8.28 ± 0.75%) observed in Trial 3 (Farm) than in Trial 2 (PHL) (Table 1).

Macrophage main effect mean levels increased (*p* < 0.001) from 0.57 ± 0.52% at 0 h to 4.37 ± 0.38% at 6 h, remained elevated at 24 h and 48 h (3.89 and 3.73 ± 0.38%, respectively) before decreasing (*p* < 0.001) to above pre-injection levels by 72 h (2.52 ± 0.38%). There were no differences in macrophage levels between treatments. However, overall macrophage levels were higher (*p* < 0.001) in Trial 2 (PHL; 3.78 ± 0.25%) than in Trial 3 (Farm; 2.24 ± 0.25%) (Table 1).

**Figure 1:**
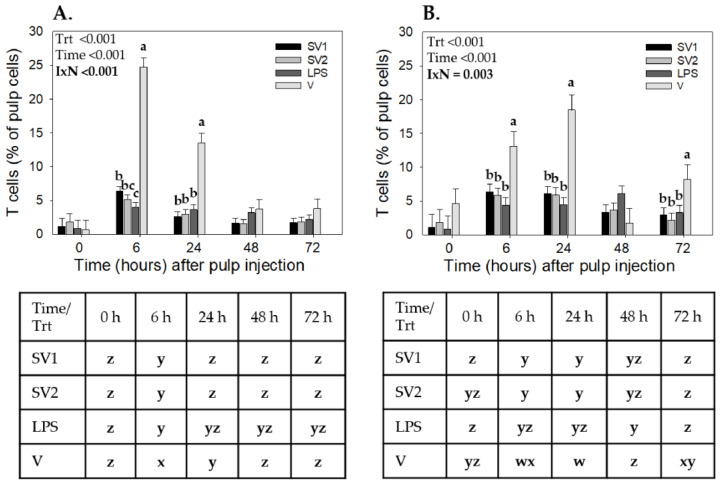
T cell infiltration profiles in response to injection of autogenous *Salmonella*-killed vaccines (SV1 and SV2), *S*. Enteritidis lipopolysaccharide (SE-LPS), or vehicle into the pulp of growing feathers. Twenty-four growing feathers (GF) of 14 to 15 wk old Light-brown Leghorn (LBL) pullets from (**A**) Trial 2-PHL and (**B**) Trial 3-Farm were injected with 10 µL of SV1, SV2, SE-LPS, or vehicle (water-oil-water emulsion). Injected GFs from each chicken were collected before injection (0 h) and at 6, 24, 48, and 72 h post-GF injection for leukocyte population analysis. Pulp cell suspensions were prepared from each GF, immunofluorescently stained with fluorescence-conjugated mouse monoclonal antibody (Southern Biotech) to chicken CD3 (T cells), and the percentage of CD3+ pulp cells was determined by fluorescence-based flow cytometry. Data shown are mean ± SEM. For each Trial, *n* = 4 pullets for SV1, SV2, and LPS, and *n* = 3 for the vehicle. Due to interactions involving the Trial, two-way ANOVA was conducted for each Trial. Student *t*-test multiple means comparisons were conducted to identify Treatment (Trt) and Time (h) differences. a–c: for each time point, treatment means without a common letter are different (*p* < 0.05); w–z: for each treatment, means at each time-point without a common letter are different (*p* < 0.05).

For T cells, the three-way ANOVA resulted in treatment, time, and trial interactions (*p* = 0.009) for T cells; therefore, data from each trial were individually analyzed using a two-way ANOVA. In Trial 2, T cells showed a treatment-by-time interaction (*p* < 0.001). All treatments resulted in T cell recruitment, with the highest levels (*p* ≤ 0.05) observed at 6 h (Figure 1A). T cell levels were highest at 6 h and 24 h in response to V injection compared to the other treatments. Specifically, with V injection, at 6 h, T cell levels were 24.7 ± 1.44%, followed by SV1 (6.35 ± 0.72%), SV2 (5.12 ± 0.72%), and SE-LPS (3.95 ± 0.72%). At 24 h, T cell levels in response to V injection declined (13.4 ± 1.44%) but remained higher (*p* < 0.001) than those of SV1 (2.57 ± 0.72%), SV2 (2.96 ± 0.72%), and SE-LPS (3.62 ± 0.72%). There were no differences in T cell levels between SV1, SV2, and SE-LPS at any of the time points examined (Figure 1A). For Trial 3, there was also a treatment-by-time interaction (*p* = 0.003) for T cells. As with Trial 2, all treatments resulted in T cell recruitment (*p* ≤ 0.05). At 6, 24, and 72 h, T cell levels were higher in response to V injection compared to SV1, SV2, and SE-LPS. There were no differences in T cell levels between SV1, SV2, and SE-LPS treatments at any of the time points examined (Figure 1B).

**Figure 2:**
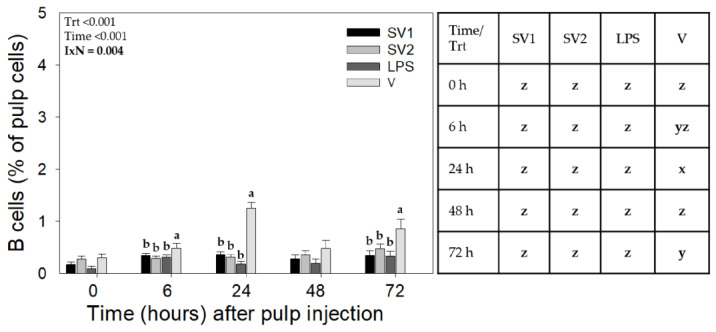
B cell infiltration profiles in response to injection of autogenous *Salmonella*-killed vaccines (SV1 and SV2), *S.* Enteritidis lipopolysaccharide (SE-LPS), or vehicle into the pulp of growing feathers. Forty-eight growing feathers (GF) of 14 to 15 wk old Light-brown Leghorn (LBL) pullets from Trial 2-PHL and Trial 3-Farm were injected with 10 µL of SV1, SV2, SE-LPS, or vehicle (water-oil-water emulsion). Injected GFs from each chicken were collected before injection (0 h) and at 6, 24, 48, and 72 h post-GF injection for leukocyte population analysis. Pulp cell suspensions were prepared from each GF, immunofluorescently stained with fluorescence-conjugated mouse monoclonal antibody (Southern Biotech) to chicken Bu-1 (B cells), and the percentage of Bu-1+ pulp cells determined by fluorescence-based flow cytometry. Data shown are mean ± SEM. For each Trial, *n* = 8 pullets for SV1, SV2, and LPS, and *n* = 6 for the vehicle. Student *t*-test multiple means comparisons were conducted to identify Treatment (Trt) and Time (h) differences. a–c: for each time point, treatment means without a common letter are different (*p* < 0.05); x–z: for each treatment, time means without a common letter are different (*p* < 0.05).

While there were no interactions involving Trial, treatment-by-time interactions (*p* = 0.004) were also observed for B cell infiltration (Figure 2). At 24 and 72 h, B cell levels were higher with V (*p* ≤ 0.05) compared to SV1, SV2, and SE-LPS. B cell levels did not differ between SV1, SV2, and SE-LPS at any of the time points examined. B cell recruitment levels changed with time only in response to V injection (Figure 2).

### 3.2. S. Enteritidis (SE) Specific Antibody Levels in Plasma Following GF-Pulp Injection of SV1, SV2, SE-LPS, or Vehicle

**Figure 3:**
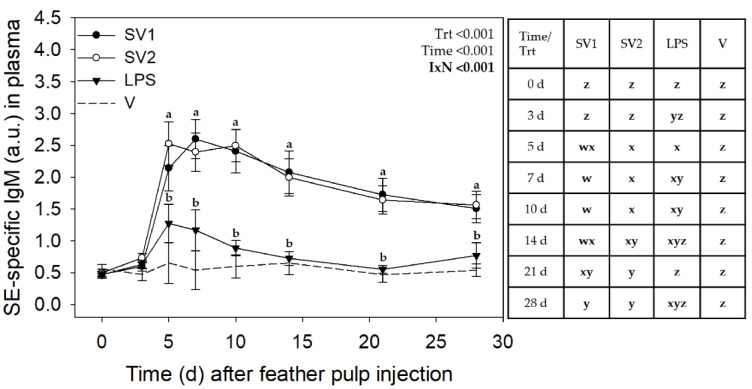
*Salmonella* Enteritidis specific IgM levels in plasma after injection of autogenous *Salmonella*-killed vaccines (SV1 and SV2), *S.* Enteritidis lipopolysaccharide (SE-LPS), or vehicle into the pulp of growing feathers. SE-specific IgM levels in the plasma of 14–15 wk old Light-brown Leghorn (LBL) pullets were measured after intradermal injection of SV1, SV2, SE-LPS, or vehicle (water-oil-water emulsion) into the pulp of growing feathers. Data shown were pooled across three Trials. Heparinized blood (1.5 mL) was collected before (0 d) and at 3, 5, 7, 10, 14, 21, and 28 d post-pulp injection. Data are means ± SEM; *n* = 12 pullets for SV1, SV2, and SE-LPS and *n* = 6 for vehicle (V). Two-way repeated measures ANOVA; Student *t*-test multiple means comparison-test was used to determine Treatment (Trt) and Time (Day) differences. a,b: for each time point, treatment means without a common letter are different (*p* < 0.05); w–z: for each treatment, time means without a common letter are different (*p* < 0.05). Note: the total SV1 and SV2 immunization doses for Trial 1 were 0.140 mL/bird, and for Trial 2 & 3, 0.165 mL/bird. The total SE-LPS dose was 5 µg/bird for all Trials.

**Figure 4:**
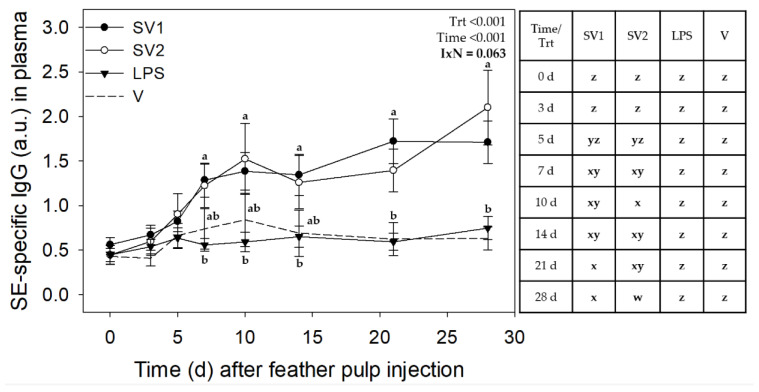
*Salmonella* Enteritidis specific IgG levels in plasma after injection of autogenous *Salmonella*-killed vaccines (SV1 and SV2), *S*. Enteritidis lipopolysaccharide (SE-LPS), or vehicle into the pulp of growing feathers. SE-specific IgG levels in the plasma of 14–15 wk old Light-brown Leghorn (LBL) pullets were measured after intradermal injection of SV1, SV2, SE-LPS, or vehicle (water-oil-water emulsion) into the pulp of growing feathers. Data shown were pooled across three Trials. Heparinized blood (1.5 mL) was collected before (0 d) and at 3, 5, 7, 10, 14, 21, and 28 d post-pulp injection. Data are means ± SEM; *n* = 12 pullets for SV1, SV2, and SE-LPS and *n* = 6 for vehicle (V). Two-way repeated measures ANOVA; Student *t*-test multiple means comparison-test was used to determine Treatment (Trt) and Time (Day) differences. a,b: for each time point, treatment means without a common letter are different (*p* < 0.05); w–z: for each treatment, time means without a common letter are different (*p* < 0.05). Note: the total SV1 and SV2 immunization doses for Trial 1 were 0.140 mL/bird, and for Trial 2 & 3, 0.165 mL/bird. The total SE-LPS dose was 5 µg/bird for all Trials.

**Figure 5:**
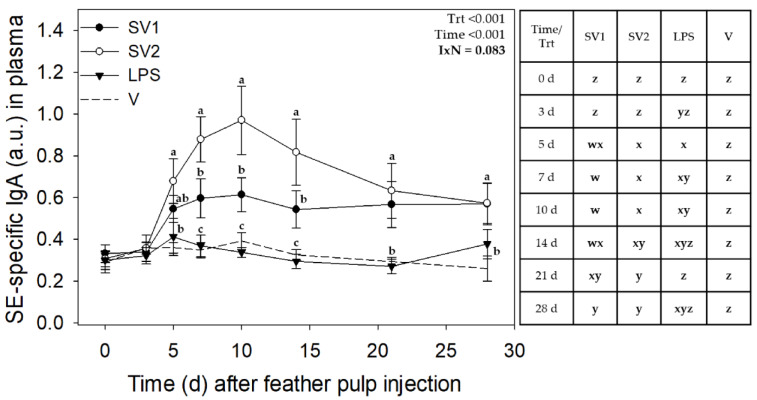
*Salmonella* Enteritidis specific IgA levels in plasma after injection of autogenous *Salmonella*-killed vaccines (SV1 and SV2), *S.* Enteritidis lipopolysaccharide (SE-LPS), or vehicle into the pulp of growing feathers. SE-specific IgA levels in the plasma of 14–15 wk old Light-brown Leghorn (LBL) pullets were measured after intradermal injection of SV1, SV2, SE-LPS, or vehicle (water-oil-water emulsion) into the pulp of growing feathers. Data shown were pooled across three trials. Heparinized blood (1.5 mL) was collected before (0 d) and at 3, 5, 7, 10, 14, 21, and 28 d post-pulp injection. Data are means ± SEM; *n* = 12 pullets for SV1, SV2, and SE-LPS and *n* = 6 for vehicle (V). Two-way repeated measures ANOVA; Student *t*-test multiple means comparison-test was used to determine Treatment (Trt) and Time (Day) differences. a–c: for each time point, treatment means without a common letter are different (*p* < 0.05); w–z: for each treatment, time means without a common letter are different (*p* < 0.05). Note: the total SV1 and SV2 immunization doses for Trial 1 were 0.140 mL/bird, and for Trial 2 & 3, 0.165 mL/bird. The total SE-LPS dose was 5 µg/bird for all Trials.

Analysis of SE antibody data from the three trials revealed no interactions involving Trial, but treatment by time interactions for IgM (*p* < 0.001) and marginal interaction for IgG (*p* = 0.063) and IgA (*p* = 0.083) (Figure 3, Figure 4 and Figure 5, respectively).

#### 3.2.1. SE-Specific IgM Antibodies in Plasma

For SV1, relative levels (a.u.) of SE-specific plasma IgM increased drastically on day 5 from 0.46 ± 0.21 before GF injection to 2.22 ± 0.21 (*p* ≤ 0.05), plateaued near this level through to 14 d, and gradually decreased (*p* ≤ 0.05) to above pre-injection levels by 28 d (1.49 ± 0.21). Similarly, for SV2, there was an increase (*p* ≤ 0.05) in SE-IgM antibody levels from 0.52 ± 0.21 before injection to peak levels at 2.54 ± 0.21 on day 5. IgM levels then gradually decreased (*p* ≤ 0.05) to 1.66 ± 0.21 by 21 d and remained above pre-injection levels on day 28 (1.58 ± 0.32). IgM increased with SE-LPS injection from 0.47 ± 0.21 on day 0 to 1.32 ± 0.21 on day 5 and returned to intermediate levels for the remainder of the 28-day sampling period. As expected, SE-specific IgM antibody levels did not change in response to V injection. In addition to different time-course profiles, IgM levels were higher (*p* ≤ 0.05) with SV1 and SV2 compared to V between 5 d and 21 d and higher than SE-LPS between 5 d and 14 d (Figure 3).

#### 3.2.2. SE-Specific IgG Antibodies in Plasma

GF injection of test materials induced SE-specific IgG with plasma levels affected by treatment (*p* < 0.001), time (*p* < 0.001), and a marginal treatment by time interaction (*p* = 0.0628) (Figure 4). For SV1, relative levels of SE-specific plasma IgG increased (*p* ≤ 0.05) from 0.40 ± 0.21 on day 0 to 1.62 ± 0.21 by 7 d and continued to gradually increase to highest levels at 21 d and 28 d. IgG levels for SV2 increased similarly over time as SV1, except for a further increase (*p* ≤ 0.05) on day 28. There were no differences in IgG levels between SV1 and SV2 at any of the time points examined. SE-specific plasma IgG levels did not change in response to SE-LPS or V injection. In addition to different time-course profiles, IgG levels tended to be higher (*p* ≤ 0.05) with SV1 and SV2 than with SE-LPS and the V from 7 d onwards. However, this was not always statistically (*p* ≤ 0.05) significant (Figure 4).

#### 3.2.3. SE-Specific IgA Antibodies in Plasma

SE-specific IgA antibody levels in plasma were affected by treatment (*p* < 0.001) and time (*p* < 0.001), with a marginal treatment by time interaction (*p* = 0.082) (Figure 5). For SV1 and SV2, IgA levels were elevated on day 5, with SV1 reaching maximal levels on day 7 (0.61 ± 0.12) and SV2 on day 10 (0.96 ± 0.13), respectively; they remained near this level until 14 d, and above pre-injection levels, at 21 and 28 d. In response to SE-LPS, IgA levels were elevated on days 3–10 and near baseline levels thereafter. IgA levels were higher with SV2 on days 7–14. There was no change in IgA levels in response to V injection (Figure 5).

## 4. Discussion

Different measures have been applied in the poultry industry to prevent and control *Salmonella* infection in chickens. However, some serovars, such as *S*. Enteritidis, have persisted, leading to pathogen outbreaks and impacting human health [25]. Although killed bacterial vaccines in poultry have shown promising effects on reducing pathogen load while stimulating effective protective immune responses [9,26], there is little to no information on the tissue/cellular immune responses initiated at the vaccine’s injection site. In this study, we used the GF-pulp, a skin derivative, to gain insights into the tissue/cellular responses over 72 h following a first i.d. pulp injection of commercial autogenous *Salmonella*-killed vaccines, SE-LPS, or the WOW emulsion vehicle alone. Additionally, we monitored the SE-specific IgM, IgG, and IgA plasma levels over 4 weeks following the i.d. GF-pulp administration of the test materials [20,21,22]. To our knowledge, this research study constitutes the first comprehensive evaluation of local inflammatory responses and systemic antibody responses to the first intradermal vaccination with autogenous *Salmonella*-killed vaccines and vaccine components over time in the same individuals. Therefore, this study provides foundational knowledge on cellular and humoral immune responses to *Salmonella*-killed vaccines, serving as a reference for future studies on vaccine design and formulation to manage and prevent *Salmonella* infection in poultry.

### 4.1. Local Inflammatory Response to Intradermal Injection of Autogenous Salmonella Killed Vaccines and Vaccine Components into GF-Pulps

Following i.d. GF-pulp injections, the *Salmonella*-killed vaccines and their components were sensed by the immune system, resulting in rapid activation of local leukocytes and the recruitment of leukocytes from the blood into the injected tissue. All test materials (SV1, SV2, SE-LPS, and the WOW emulsion vehicle) induced similar recruitment of leukocytes (% pulp cells) into GF-pulps, reaching peak levels at 6 h. Leukocyte levels then gradually declined but remained above pre-injection levels by 72 h. Further leukocyte population analysis revealed that heterophils, the avian counterpart of neutrophils, were the first and most abundant cell type to be recruited to the GF-pulp. Interestingly, injection with SV1, SV2, and SE-LPS resulted in similar heterophil recruitment, which was higher than with the vehicle alone. The observation that SE-LPS stimulated heterophil infiltration similar to SV1 and SV2 indicates that LPS, and not the vehicle, is the major inflammatory component stimulating heterophil recruitment in the *Salmonella* vaccines. Additionally, the higher heterophil recruitment into injected GF-pulps in Trial 3-Farm than in Trial 2-PHL also suggests a role of LPS in the heterophil recruitment, presumably through greater exposure and likely sensitization of the innate immune system to LPS in the conventional farm environment [27].

The monocyte/macrophage infiltration also reached maximal levels by 6 h and remained at this level until 72 h post-injection, when levels dropped to proportions still higher than before injection. There were no treatment differences between SV1, SV2, SE-LPS, and the vehicle regarding the recruitment of macrophages to the injection site, demonstrating that the vehicle alone is very effective in stimulating macrophage recruitment. In a recent study, i.d., GF-pulp injection of SE-killed vaccine prepared in an endotoxin-free PBS vehicle resulted in macrophage recruitment like that observed here for all test materials, while the PBS vehicle alone did not elevate macrophage levels (unpublished data). Hence, it is not likely that the WOW emulsion vehicle is the only driving force for macrophage recruitment when mixed with SE-LPS, SV1, or SV2. Notably, macrophage infiltration was higher in Trial 2-PHL than in Trial 3-Farm, independent of treatment. This environmental effect on macrophage recruitment is opposite to the effects on heterophil recruitment, where LPS seems to be the major stimulator.

Overall, the inflammatory leukocyte recruitment profiles were as one would expect in response to the first administration of bacteria, where phagocytes like heterophils and macrophages play an important role in their elimination. Moreover, the rapid and high infiltration of heterophils within hours and their drop thereafter aligns with their role as first but short-lived responders. Heterophils have been shown to phagocytose opsonized and non-opsonized *Salmonella* [28]. The killing of this pathogen by heterophils is mediated by an oxidative burst, the cellular degranulation of bactericidal components, and net formation [10,28,29]. As part of the inflammatory activity initiated by the i.d. GF-pulp injection of test materials, monocytes infiltrated the tissue along with heterophils, albeit at much lower levels. Monocytes recruited from the blood differentiate into macrophages and exhibit enhanced phagocytosis, manufacture microbicidal factors, along with pro-inflammatory and anti-inflammatory cytokines, remove cellular debris, and promote tissue repair [30,31,32,33,34]. The sustained high levels of macrophages over the 72 h test period reflect their role in both the initiation and resolution of the inflammatory response.

Interestingly, after the injection of test materials, the recruitment of T cells into GF-pulp was different between treatments and Trials. In Trial 2-PHL, the highest levels of T cells were observed 6 h post-GF-pulp injections with all test materials, whereby the WOW emulsion vehicle recruited approximately 3–4 times more T cells than SV1, SV2, and SE-LPS. T cell levels in vehicle-injected GF-pulps remained elevated at 24 h but had already returned to pre-injection levels for SV1, SV2, and SE-LPS. For Trial 3-Farm, the dynamics of T cell recruitment were different from Trial 2-PHL in that T levels were elevated at 6 h and 24 h for all treatments, with the highest levels in response to vehicle injection. Additionally, at 48 h, T cells had dropped to pre-injection levels in vehicle-injected GF-pulps but increased again at 72 h, whereas T cells had not fully returned to baseline until 72 h post-GF-pulp injection for the other test materials. Independent of the Trial, B cells were only recruited with the vehicle injection, increasing to the highest levels by 24 h, then dropping to pre-injection levels by 48 h and increasing again at 72 h. It is not surprising that only a few T and B lymphocytes were recruited to the site of SV1, SV2, and SE-LPS administrations as this was the first exposure to *Salmonella* components, and hence the administration was expected to stimulate an innate, not antigen-specific, inflammatory response. It is difficult to speculate which component in the commercial WOW emulsion vehicle attracted lymphocytes to the site of injection. Water-oil-water emulsion vehicles are commonly used as adjuvants, affording the slow release of the antigen and producing a high and long stimulation of the immune response [35,36,37,38]. The commercial WOW emulsion vehicle used here clearly stimulated lymphocyte, monocyte/macrophage, and heterophil recruitment to the injection site. However, when mixed with the killed *Salmonella* or SE-LPS, the lymphocyte recruitment effect was greatly suppressed, whereas heterophil infiltration substantially increased. The altered leukocyte infiltration profiles in the presence of killed *Salmonella* are likely due to signals from the multiple microbe-associated molecular patterns (MAMPs), including LPS, that dominate the direction of the local innate immune response, favoring the recruitment of heterophils.

In this study, SV1 and SV2 were administered at three concentrations, i.e., undiluted (10^8^ cells/mL), diluted 1 in 2, or 1 in 4 in WOW emulsion vehicle, in different GF-pulps of the same bird. Similarly, SE-LPS was also administered at three concentrations, i.e., undiluted (180,000 EU/mL), 1 in 5, and 1 in 10 dilutions. These SE-LPS concentrations covered the range of LPS content of the SV1 and SV2 dilutions tested, e.g., from 1800 EU (0.36 µg) LPS to 180 EU (0.036 µg) LPS per GF-pulp. Statistical analysis revealed no dosage effect for any of the leukocyte recruitment profiles examined. This was not surprising as a previous dose-response study revealed similar leukocyte infiltration profiles when GF-pulps of juvenile egg-type chickens were injected with 1, 0.1, or 0.01 µg *Salmonella* Typhimurium (ST) LPS [39]. It should be noted that ST-LPS was prepared in endotoxin-free PBS, and, most importantly, each dose was tested in separate chickens (not in the same chicken as in the current study) in that study. Overall, our results indicate that LPS alone appears to be as effective as the whole killed *Salmonella* in triggering an inflammatory response in the injected tissue, exhibiting similar characteristics over a range from 0.036 to 0.36 µg/GF-pulp.

### 4.2. Plasma SE-Specific IgM, IgG, and IgA Levels after Intradermal Injection of Autogenous Salmonella Killed Vaccines and Vaccine Components into GF-Pulps

While lymphocytes did not play a major part in the local inflammatory response initiated by the *Salmonella* vaccines, the vaccines in the WOW emulsion vehicle did stimulate an antigen-specific antibody response, presumably due to effective antigen-presentation and stimulation of the adaptive immune response in secondary lymphoid organs, which in chickens likely occurred in the spleen [40]. This is supported by the temporal, quantitative, and qualitative aspects of SE-specific antibody profiles in the peripheral blood circulation over 4 weeks post-i.d. GF-pulp injection of SV1, SV2, or SE-LPS. As SE was the dominant serotype in both autogenous *Salmonella*-killed vaccines, plasma levels of SE-specific IgM, IgG, and IgA antibodies were determined by ELISA to gain insight into the humoral responses to the *Salmonella* vaccines and SE-LPS. SV1 and SV2 produced similar SE-specific IgM and IgG antibody production profiles, with IgM reaching maximal levels (a.u.) on days 5 to 10 post-i.d. GF-pulp injection. IgM levels then gradually declined but remained above pre-injection levels throughout 28 d. Plasma SE-specific IgG levels were elevated above pre-injection levels by 7 d and continued to gradually increase reaching maximal levels on day 21 and 28. The early IgM peak and the later sustained increase in IgG are typical of a primary T-dependent antibody response, where antigen-activated helper T cells drive the isotype switch from IgM to IgG [41]. Similarly, both SV1 and SV2 resulted in SE-specific IgA production, with elevated plasma levels from 5 d onward. Specifically, plasma SE-specific IgA levels were highest between 5 d and 14 d, then declined gradually, remaining above pre-injection levels on day 28. While the time course trend was similar for SV1 and SV2, SE-specific IgA levels in plasma were lower between 5 d and 14 d with SV1 compared to SV2. It is not clear why more SE-IgA was produced in response to SV2 as the exact composition of the autogenous killed vaccines is not known; it is possible that SE, albeit the most abundant bacterium in both vaccines, was present at lower proportions and, hence, lower numbers, in SV1 than in SV2.

The relative plasma levels of SE-specific IgA were lower than those of SE-specific IgG, suggesting preferential isotype switch to IgG. For systemic protection, IgG is a high-quality antibody in the fight against *Salmonella* infection, capable of opsonization, enhancing phagocytosis and microbicidal activities of phagocytes, complement activation, and antibody-dependent cellular cytotoxicity, and it serves as a maternal antibody [41,42,43]. IgA, on the other hand, will have protective effects as secretory IgA by neutralizing *Salmonella* and preventing infection at the mucosal surfaces [44,45]. For example, some studies have associated *Salmonella* clearance in the chicken’s gut with increased serum IgA levels [46,47]. Circulating IgA levels are, however, not a clear reflection of IgA production by plasma cells in mucosal tissues and secretion of IgA across the mucosal epithelium.

Overall, both SV1 and SV2 stimulated a reliably high-quality, primary antibody response to SE, a major component of both autogenous killed vaccines. As this primary antibody response is characteristic of a T-dependent response, the autogenous killed-vaccine formulation and the WOW emulsion vehicle are effective in the activation and differentiation of a T cell response. It will be interesting to examine secondary response profiles to gain insight into effector T cell differentiation, immunological memory development, and affinity maturation. Moreover, considering that live *Salmonella* is an intracellular pathogen, future studies should examine the cell-mediated immunity generated by these vaccines.

SE-LPS administration also resulted in an increase in SE-specific IgM, whereby plasma levels were only elevated between 5 d and 10 d and at substantially lower levels than with SV1 and SV2. For LPS, there was no SE-specific IgG and IgA response in peripheral blood over the 28-day post-injection period. The observed early, short-lived IgM response without a switch to IgG is typical of the response to a T-independent antigen such as LPS (lipopolysaccharide), which does not have a protein component needed to stimulate, antigen-specific help from αβ TCR CD4+ helper T cells [41,42,43,48].

Previously, a study conducted in non-sensitized specific pathogen-free (SPF) chickens found that the serum IgM, IgG, and IgA specific to *Salmonella* Typhimurium (ST) increased soon after a first challenge exposure, peaked at 13 d with IgM gradually decreasing thereafter, yet serum ST-specific IgG and IgA levels remained elevated by 28 d post-injection [48]. This trend of a classical antibody response to a primary infection of *Salmonella* in SPF chickens is in agreement with our observations, further supporting that the autogenous *Salmonella* bacterin vaccines can induce a robust, systemic antigen-specific antibody response in egg-type chickens.

## 5. Conclusions

Three key insights were drawn from assessing the local tissue leukocyte recruitment profiles and systemic SE-specific IgM, IgG, and IgA antibody responses following i.d. GF-pulp injection with autogenous *Salmonella*-killed vaccines and their components. (A) The inflammatory response initiated by a first injection of SV1 and SV2 involved the recruitment of phagocytes, especially heterophils and monocytes/macrophages, with minor participation of lymphocytes. This innate inflammatory response was near a complete resolution by 72 h and was similar to that observed with SE-LPS. (B) The WOW emulsion vehicle alone recruited fewer heterophils but substantially more T and B lymphocytes than SV1, SV2, and SE-LPS; this effect was, however, suppressed when the vehicle was mixed with *Salmonella*-killed bacteria or SE-LPS. (C) SV1 and SV2 stimulated a robust systemic, primary, SE-specific T-dependent antibody response, with the characteristic switch from SE-specific IgM to IgG in the later phase of the response. It should be noted that this robust antibody response was generated by i.d. GF-pulp injection of only one-third of the recommended dose for subcutaneous or intramuscular administration of the autogenous *Salmonella*-killed vaccine. Overall, the insights gathered from this research study provide a comprehensive perspective into the innate and adaptive immune responses to i.d. administration of autogenous *Salmonella*-killed vaccines in egg-type chickens, which could assist in developing more effective *Salmonella* vaccines for poultry.

## Figures and Tables

**Table 1:** Leukocyte, heterophil, and macrophage levels (% of pulp cells) in growing feathers of Light-brown Leghorn pullets following injection of SV1, SV2, SE-LPS, or vehicle in two trials.

Time (h) ^2^	Leukocytes ^1^	Heterophils ^1^	Macrophages ^1^
0	5.4 ± 2.24 d	1.3 ± 1.49 d	0.6 ± 0.49 c
6	38.7 ± 1.64 a	20.1 ± 1.09 a	4.4 ± 0.36 a
24	29.6 ± 1.64 b	13.7 ± 1.09 b	3.9 ± 0.36 a
48	18.2 ± 1.64 c	7.0 ± 1.09 c	3.7 ± 0.36 a
72	15.0 ± 1.64 c	5.4 ± 1.09 c	2.5 ± 0.36 b
**Treatment ^3^**			
SV1	22.1 ± 1.31	11.0 ± 0.87 a	3.2 ± 0.29
SV2	20.4 ± 1.31	9.7 ± 0.87 a	3.1 ± 0.29
SE-LPS	21.2 ± 1.31	11.4 ± 0.87 a	2.8 ± 0.29
Vehicle	21.8 ± 2.22	5.9 ± 0.87 b	3.0 ± 0.49
**Trial ^4^**			
2-PHL	20.7 ± 1.12	8.3 ± 0.75 b	3.8 ± 0.25 a
3-Farm	22.1 ± 1.12	10.7 ± 0.75 a	2.2 ± 0.25 b
**Effects (*p*-value) ^5^**			
Treatment	0.813	**0.012**	0.710
Time	**<0.001**	**<0.001**	**<0.001**
Trial	0.380	**0.022**	**<0.001**
Treatment x Time	0.999	0.187	0.875
Treatment x Trial	0.783	0.897	0.807
Time x Trial	0.409	0.852	0.102
Treatment x Time x Trial	0.687	0.598	0.782

^1^ Percentage of leukocytes in total pulp cell suspension (% pulp cells). ^2^ Time: Before (0 h) and at 6, 24, 48, and 72 h post-pulp injection of test materials. ^3^ Treatment: The pulp of 24 growing feathers of Light-brown Leghorn pullets was injected (i.d.) with 10 μL of autogenous *Salmonella*-killed vaccine 1 (SV1), SV2, *S*. Enteritidis lipopolysaccharide (SE-LPS) or vehicle (water-oil-water emulsion); 4 pullets for SV1, SV2, and SE-LPS; 3 pullets for the vehicle in each of two trials. ^4^ Trial: Poultry Health Lab Room 4 (PHL; pullets not HVT-vaccinated) and the Poultry Farm (Farm; pullets HVT vaccinated).^5^ Three-way ANOVA; significance considered at *p* ≤ 0.05. Student *t*-test multiple means comparisons were conducted to identify treatment and time differences. a–d: within a cell type and variable, means without a common letter are different (*p* ≤ 0.05).

## Data Availability

The data presented in this study are available on request from the corresponding authors.
